# Impact of acyclovir use on survival of patients with ventilator-associated pneumonia and high load herpes simplex virus replication

**DOI:** 10.1186/s13054-019-2701-5

**Published:** 2020-01-10

**Authors:** Lukas Schuierer, Michael Gebhard, Hans-Georg Ruf, Ulrich Jaschinski, Thomas M. Berghaus, Michael Wittmann, Georg Braun, Dirk H. Busch, Reinhard Hoffmann

**Affiliations:** 1 TUM Graduate School, Technical University of Munich (TUM), Munich, Germany; 2 Institute for Laboratory Medicine and Microbiology, University Hospital Augsburg, Stenglinstr.2, 86156 Augsburg, Germany; 3 Faculty of Medicine, Augsburg University, Augsburg, Germany; 4 Department of Radiology, University Hospital Augsburg, Augsburg, Germany; 5 Department of Anesthesiology and Surgical Intensive Care Medicine, University Hospital Augsburg, Augsburg, Germany; 6 Department of Internal Medicine I, University Hospital Augsburg, Augsburg, Germany; 7 Department of Internal Medicine II, University Hospital Augsburg, Augsburg, Germany; 8 Department of Internal Medicine III, University Hospital Augsburg, Augsburg, Germany; 9 Institute for Medical Microbiology, Immunology and Hygiene, Technical University of Munich, Munich, Germany

**Keywords:** Ventilator-associated pneumonia, Bronchoalveolar lavage fluid, Simplexvirus, Real-time polymerase chain reaction, Acyclovir

## Abstract

**Background:**

Herpes simplex virus (HSV) replication can be detected in the respiratory secretions of a high proportion of ventilated intensive care unit (ICU) patients. However, the clinical significance remains poorly defined. We investigated whether patients with ventilator-associated pneumonia not responding to antibiotics and in whom high levels of HSV could be detected in respiratory secretions benefit from acyclovir treatment.

**Methods:**

Respiratory secretions (bronchoalveolar lavage fluid or tracheal aspirates) were tested for HSV replication by quantitative real-time PCR. ICU survival times, clinical parameters, and radiographic findings were retrospectively compared between untreated and acyclovir treated patients with high (> 10^5^ HSV copies/mL) and low (10^3^–10^5^ HSV copies/mL) viral load.

**Results:**

Fifty-seven low and 69 high viral load patients were identified. Fewer patients with high viral load responded to antibiotic treatment (12% compared to 40% of low load patients, *p* = 0.001). Acyclovir improved median ICU survival (8 vs 22 days, *p* = 0.014) and was associated with a significantly reduced hazard ratio for ICU death (HR = 0.31, 95% CI 0.11–0.92, *p* = 0.035) in high load patients only. Moreover, circulatory and pulmonary oxygenation function of high load patients improved significantly over the course of acyclovir treatment: mean norepinephrine doses decreased from 0.05 to 0.02 μg/kg body weight/min between days 0 and 6 of treatment (*p* = 0.049), and median PaO_2_/FiO_2_ ratio increased from 187 to 241 between day 3 and day 7 of treatment (*p* = 0.02). Chest radiographic findings also improved significantly (*p* < 0.001).

**Conclusions:**

In patients with ventilator-associated pneumonia, antibiotic treatment failure, and high levels of HSV replication, acyclovir treatment was associated with a significantly longer time to death in the ICU and improved circulatory and pulmonary function. This suggests a causative role for HSV in this highly selected group of patients.

## Background

Latent herpes simples virus type 1 (HSV-1) and type 2 (HSV-2) infections are common in the human population, with seroprevalence rates of 54% and 16%, respectively [1]. Residing latently in sensory neurons, HSV reactivates in states of reduced immunocompetence [2]. Several studies have shown that HSV-1 reactivation and active replication in the respiratory tract are common in mechanically ventilated intensive care unit (ICU) patients even without underlying immunosuppression, with reported rates of 5 to 64% [3]. Ong et al., in the largest study available to date, detected active HSV replication in 27% of 393 ventilated ICU patients, which was associated with a nearly twofold increase in hospital mortality (41% vs 24%, *p* = 0.002) [4].

Nevertheless, whether HSV replication in the lower respiratory tract has clinical consequences remains controversial [5, 6]. Linssen et al. reported that detection of more than 10^5^ HSV-DNA copies/mL in lower respiratory material was associated with a significantly higher mortality (41% vs 20%, *p* = 0.001) [7]. A recent meta-analysis demonstrated a significant increase in mortality (odds ratio 1.8, 95% CI 1.2–2.6, *p* = 0.0001) for patients with HSV replication compared to patients without [8]. Whether this increase in mortality is caused by HSV reactivation or whether HSV reactivation is merely an indicator of a more severe underlying disease remains unclear.

Similarly, cytomegalovirus (CMV) reactivation can be detected in respiratory secretions of about 16% immunocompetent ICU patients [9]. Much like in HSV, the clinical consequences remain unclear.

Some case reports suggest that acyclovir treatment may improve the clinical course of patients with detectable HSV reactivation [10–13]; however, this could not be confirmed in larger studies [14–16]. To date, only one study has suggested that acyclovir may reduce in-hospital and ICU mortality, even after correcting for confounders via propensity score matching [17]. The reasons for these conflicting results remain unclear; however, it is apparent that most of the previous studies examined HSV replication, corresponding treatment, and the impact on survival among a diverse range of ICU patients. Often, stringent inclusion criteria for presumed viral ventilator-associated pneumonia (VAP) were not employed, and patients were not stratified for viral load. In fact, many patients with molecularly detectable HSV replication may not have clinical signs of pneumonia, precluding any conclusions regarding the therapeutic efficacy of acyclovir.

We observed that in some patients with high levels of HSV replication in the lower respiratory tract, the clinical status improved after initiation of antiviral treatment, often allowing extubation several days later. Therefore, we changed our treatment algorithm for patients with VAP early in 2013. In patients with clinical signs of pneumonia who did not respond to antibiotic treatment (as determined in joint rounds by clinicians and experienced clinical microbiologists performed routinely three times per week), HSV-1/2 testing of respiratory specimens was performed. Antiviral treatment was strongly encouraged if more than 10^5^ HSV copies/mL could be detected. However, final treatment decisions were left to the responsible physician. We hypothesized that these stringent selection criteria would result in a significantly more homogeneous patient population than in previous studies, with a higher pretreatment likelihood of HSV pneumonia, and would, for the first time, allow for direct evaluation of acyclovir treatment efficacy in these patients.

## Methods

### Patient selection and PCR testing

We retrospectively identified all adult ICU patients who were on ventilator support, received a diagnosis of VAP and PCR testing on clinical grounds (as determined jointly by clinicians and clinical microbiologists, based on elevated C-reactive protein, leukocytosis, purulent tracheal secretions, infiltrates on chest X-ray, or typical signs in bronchoscopy), and had a viral load of > 10^3^ HSV-1/2 copies/mL by PCR in respiratory specimens (excluding oropharyngeal swabs from herpes-suspected lesions) within the period of January 1, 2013, through April 1, 2018. We defined the time point of VAP diagnosis as the first radiological demonstration of pulmonary infiltrates. In those patients without clear-cut infiltrates, a diagnosis of VAP was made based on bronchoscopy findings. Neutropenic patients (neutrophils < 1500/μL) were excluded. Response to antibiotic treatment was assessed during regular daily ICU rounds, usually 48 h after initiation; responding patients were excluded from the analysis. Quantitative HSV-1/2 PCR was performed after automated DNA extraction by COBAS AmpliPrep (Roche) with the RealStar HSV-PCR kit 1.0 (AltonaDiagnostics) on a Rotor-Gene-Q thermocycler (Qiagen) according to the manufacturer’s recommendations. Additional CMV testing was performed as required by the treating physician. Quantitative CMV PCR was performed with the COBAS AmpliPrep/COBAS TaqMan CMV test (Roche) according to the manufacturer’s recommendations.

### Clinical and radiographic scores

Day 0 (d0) was defined as the date of first detection of significant HSV-1/2 replication (low load, 10^3^–10^5^ copies/mL; high load, > 10^5^ copies/mL) in untreated patients or as the date of acyclovir treatment start for patients receiving acyclovir. Data were evaluated from d-4 up to d+14.

The Charlson Comorbidity Index was determined on d0 based on the treating physician’s discharge letters [18]. The “Acute Physiology, Age, Chronic Health Evaluation II” (APACHE II) score and “Sequential Organ Failure Assessment” (SOFA) score were calculated on d0 and with the most abnormal values in first 24 h after ICU admission, as described [19, 20]. The Glasgow Coma Score was used as documented by responsible ICU staff in the patient charts.

Chest X-rays and lung computed tomography (CT) were taken based on individual clinical necessity as assessed by treating ICU physicians. The standardized Lung Injury Score (LIS) and the simplified version of the Clinical Pulmonary Infection Score (CPIS) were calculated as described [21, 22]. Imaging results from all patients with a high viral load were re-analyzed in a standardized way to quantitatively assess appearance and distribution of pulmonary infiltrates over time. Based on the descriptive radiologic part of the LIS score, we assigned points to each of the six lung fields (upper/middle/lower, left, and right, respectively), with 1 point = “no infiltrates,” 2 points = “discrete/questionable infiltrate,” and 3 points = “distinct infiltrate.” We evaluated our score by re-analyzing all radiographs using the descriptive radiologic part of the LIS score with essentially identical results (Additional file 1: Figure S3).

### Clinical and laboratory parameters

**Fig. 1:**
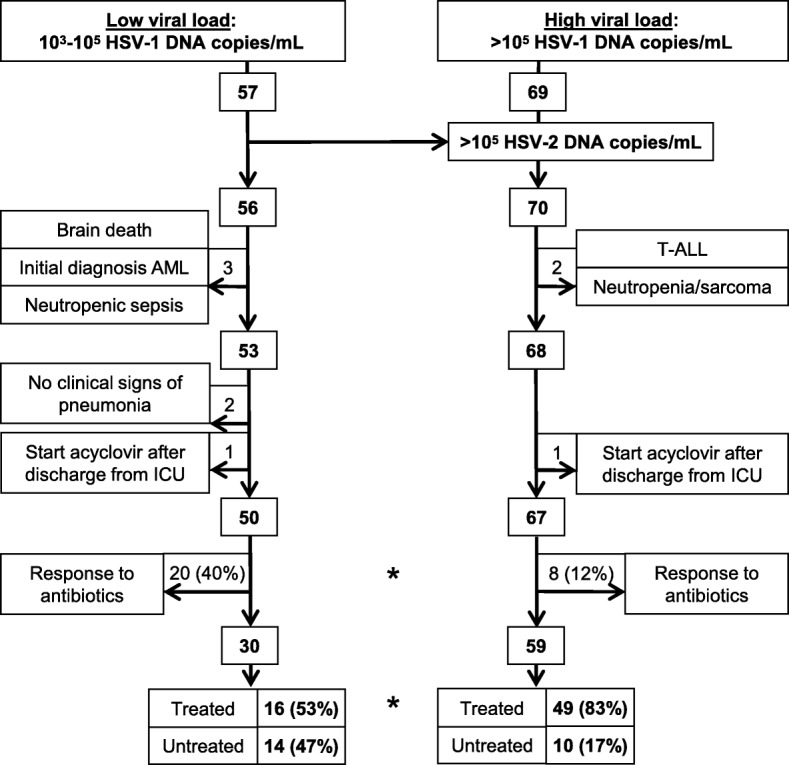
Enrollment of patients with ventilator-associated pneumonia according to low or high viral load between 2013 and 2018: intensive care unit (ICU) patients on ventilator support received a quantitative real-time polymerase chain reaction (PCR) testing of their respiratory material (bronchoalveolar lavage or tracheobronchial aspirates) in context of a ventilator-associated pneumonia. Figure indicates numbers of patients; figures next to outward pointing arrows show excluded patients. Reasons for exclusion are indicated in boxes. HSV-1, herpes simplex virus type 1; HSV-2, herpes simplex virus type 2; AML, acute myelogenous leukemia; T-ALL, T cell acute lymphoblastic leukemia. *Statistically significant with *p* < 0.05 by Fisher’s exact test

**Fig. 2:**
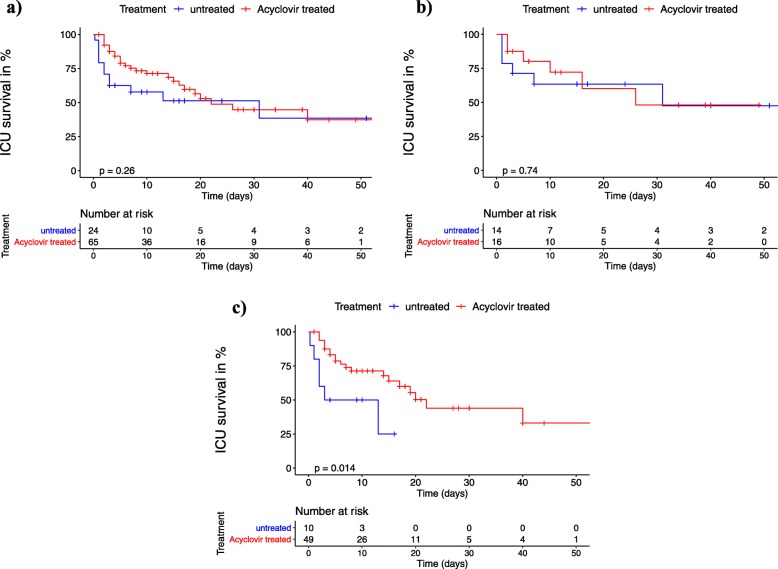
**a**–**c** Kaplan-Meier analysis of intensive care unit survival after starting antiviral treatment or after HSV detection: day 0 (d0) was defined as the date of first detection of significant HSV-1/2 replication in untreated patients or as the date of acyclovir treatment start for patients receiving. + = censored. The *p* values were calculated using a log-rank test. **a** Entire cohort. **b** Subgroup with low viral load (10^3^–10^5^ HSV copies/mL). **c** Subgroup with high viral load (> 10^5^ HSV copies/mL)

**Fig. 3:**
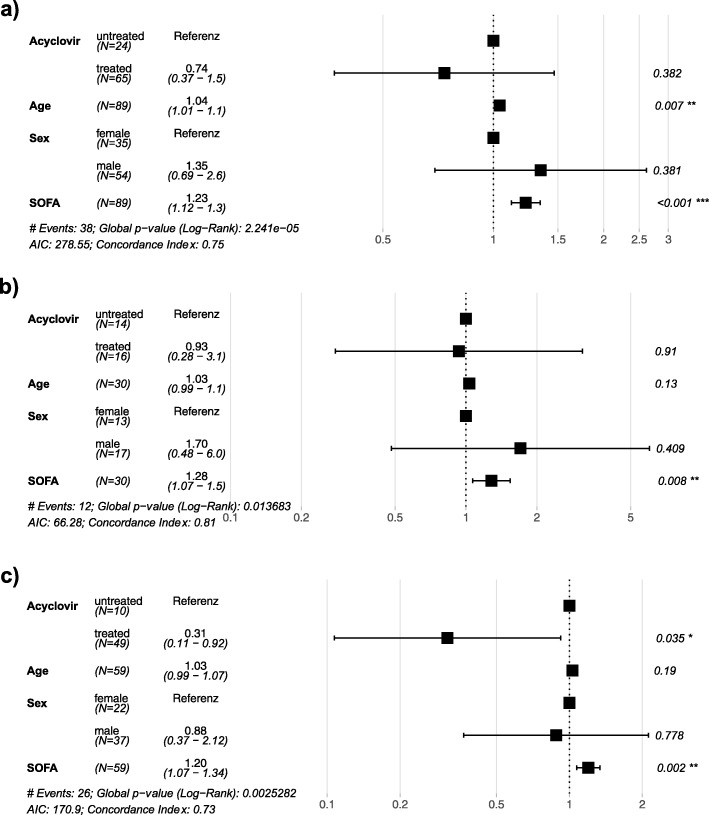
**a**–**c** Hazard ratios for ICU death from the adjusted multivariable Cox model: the multivariable Cox model was adjusted for age, sex, and SOFA score at d0. The hazard ratios are labeled on the *x*-axis. The horizontal bars are 95% confidence intervals (95% CI), and significant (< 0.05) *p* values are indicated with asterisk. **a** Entire cohort. **b** Subgroup with low viral load (10^3^–10^5^ HSV copies/mL). **c** Subgroup with high viral load (> 10^5^ HSV copies/mL)

**Fig. 4:**
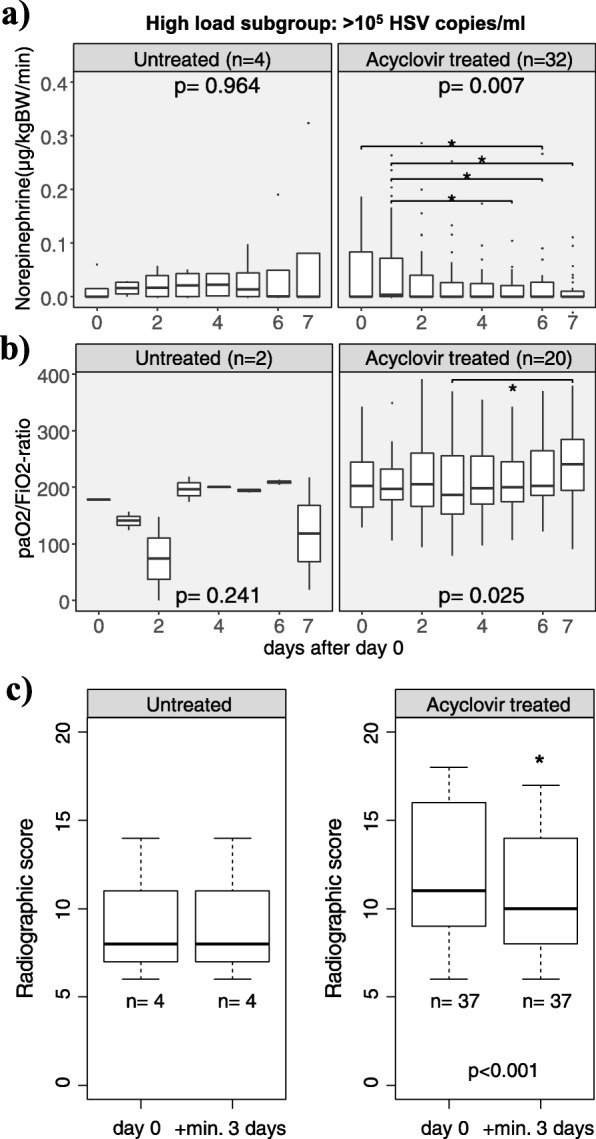
**a**–**c** Development of clinical parameters in high viral load patients after start of antiviral treatment. Day 0 (d0) was defined as the date of first detection of significant HSV-1/2 replication in untreated patients or as the date of acyclovir treatment start for patients receiving. **a** Circulatory function as measured by norepinephrine doses given for circulatory support. **b** Respiratory function as measured by PaO_2_/FiO_2_ ratio. **c** Pulmonary infiltrates as measured by a semiquantitative score. In **a** and **b**, *p* values are from the Friedman test, while horizontal brackets indicate significant differences (*p < 0.05) between time points by Conover’s post hoc test. In **c**, the Wilcoxon signed-rank test was used to compare the last available X-ray or CT before d0 to the maximum change within a time span of 3 to 15 days

The clinical and laboratory parameters listed in Table 1 and Figs. 1, 2, 3, and 4 were extracted from the hospital information systems (Hydmedia G5 and Orbis, Agfa), from the laboratory information system (Swisslab, Nexus AG, Donaueschingen, Germany), or manually from handwritten ICU charts at different time points.

**Table 1:** Baseline clinical and treatment characteristics of the entire cohort and the subgroups (low/high viral load)

Variables	All patients			Low viral load (10^3^–10^5^ HSV copies/mL)			High viral load (> 10^5^ HSV copies/mL)		
	Untreated (*n* = 24)	Treated (*n* = 65)	*p*	Untreated (*n* = 14)	Treated (*n* = 16)	*p*	Untreated (*n* = 10)	Treated (*n* = 49)	*p*
Age (years)	72 [67–76]	69 [59–76]	0.274	69 [61–77]	65 [46–75]	0.308	73 [71–76]	71 [60–76]	0.187
Female	12 (50)	23 (35)	0.230	9 (64)	4 (25)	0.063	3 (30)	19 (39)	0.729
Charlson score	4 [3–6]	4 [2–6]	0.281	4 [3–6]	3 [3–5]	0.179	4 [3–6]	4 [2–6]	0.743
Intubation (days)	9 [6–12]	13 [8–17]	*0.017*	9 [6–12]	12 [7–18]	0.324	10 [7–11]	14 [9–17]	0.109
Lung disease	15 (62)	31 (48)	0.241	9 (64)	10 (63)	1	6 (60)	21 (43)	0.488
COPD	12 (50)	13 (20)	*0.008*	7 (50)	2 (13)	*0.046*	5 (50)	11 (22)	0.116
Active smoker	6 (25)	18 (28)	1	4 (29)	5 (31)	1	2 (20)	13 (27)	1
Dialysis	7 (29)	19 (29)	1	5 (36)	7 (44)	0.722	2 (20)	12 (24)	1
Diabetes	6 (25)	13 (20)	0.771	4 (29)	3 (19)	0.675	2 (20)	10 (20)	1
Malignant diseases	3 (12)	10 (15)	1	2 (14)	2 (13)	1	1 (10)	8 (16)	1
Quantitative polymerase chain reaction results									
BAL performed	18 (75)	45 (69)	0.793	12 (86)	13 (81)	1	6 (60)	32 (54)	0.733
HSV—copies/mL ×10^5^	0.13 [0.05–2.18]	10.78 [1.01–60.15]	*< 0.001*	0.06 [0.04–0.09]	0.16 [0.04–0.40]	0.085	2.84 [1.80–6.80]	30.69 [7.26–12.42]	*< 0.001*
BAL: HSV—copies/mL ×10^5^	0.09 [0.04–1.22]	10.50 [0.41–41.55]	*< 0.001*	0.05 [0.04–0.09]	0.13 [0.03–0.27]	0.205	2.37 [1.45–2.89]	28.30 [6.77–83.50]	*0.005*
TBS: HSV—copies/mL ×10^5^	3.19 [0.61–15.79]	19.29 [6.50–143.9]	0.108	0.14 [0.10–0.19]	0.55 [0.34–0.76]	0.400	12.08 [3.92–39.63]	44.85 [10.5–169.2]	0.247
Pulmonary infiltrates									
Infiltrates	16 (66)	54 (83)	0.143	11 (79)	13 (81)	1	5 (50)	41 (84)	*0.033*
Questionable but pathologic brochoscopy	4 (17)	5 (8)	0.244	1 (7)	1 (6)	1	3 (30)	4 (8)	0.087
No infiltrates but pathologic bronchoscopy	4 (17)	6 (9)	0.449	2 (14)	2 (13)	1	2 (20)	4 (8)	0.266
Days from detection of infiltrates to HSV detection	7 [3–11]	8 [5–14]	0.318	6 [2–8]	8 [5–13]	0.126	12 [8–15]	8 [5–14]	0.444
Clinical score at day of HSV detection									
APACHE II score	31 [25–37]	27 [22–33]	0.081	31 [25–37]	26 [21–34]	0.219	31 [28–36]	27 [22–33]	0.223
SOFA score	11 [7–13]	10 [6–11]	0.091	11 [8–14]	10 [6–11]	0.325	10 [8–13]	9 [6–11]	0.294
LIS score	2 [1.4–2.8]	2.3 [1.9–3]	*0.050*	2 [1.5–2.8]	2.2 [2.0–3.0]	0.134	1.8 [1.1–2.6]	2.3 [1.8–3]	0.123
CPIS score	5 [3–6]	5 [3–6]	0.455	5 [4–5]	4 [3–5]	0.519	6 [3–7]	5 [3–6]	0.402
Antiviral treatment and relevant medication									
Acyclovir	–	63 (97)	–	–	14 (88)	–	–	49 (100)	–
Ganciclovir	–	3 (5)	–	–	2 (13)	–	–	1 (2)	–
Hours from HSV detection to treatment	–	46 [30–68]	–	–	48 [31–95]	–	–	44 [30–67]	–
Acyclovir + antibiotics	–	44 (68)	–	–	11 (69)	–	–	33 (67)	–
Steroids at baseline	4 (17)	13 (20)	1	2 (14)	6 (38)	0.226	2 (20)	7 (14)	0.641
Antibiotic classes	4 [2–5]	5 [3–6]	*0.025*	4 [2–6]	6 [5–6]	0.051	4 [2.0–5]	5 [3–6]	0.124
Antibiotics (days)	15 [9–23]	18 [14–28]	*0.046*	20 [7–26]	26 [18–37]	0.094	12 [10–17]	17 [13–25]	*0.045*
Catecholamines	23 (96)	63 (97)	1	13 (93)	15 (94)	1	10 (100)	48 (98)	1
Catecholamines (days)	10 [5–13]	10 [7–19]	0.523	11 [4–12]	8 [6–20]	0.847	8 [6–14]	11 [8–18]	0.505
Diagnoses at ICU admission									
Respiratory insufficiency	22 (92)	58 (89)	1	14 (100)	14 (88)	0.485	8 (80)	44 (90)	0.338
Sepsis	14 (58)	26 (40)	0.153	9 (64)	9 (56)	0.722	5 (50)	17 (35)	0.477
Renal failure	9 (38)	15 (23)	0.188	7 (50)	2 (13)	*0.046*	2 (20)	13 (27)	1
Cardiac arrest	2 (8)	4 (6)	0.659	1 (7)	1 (6)	1	1 (10)	3 (6)	0.535
Reasons for mechanical ventilation									
Sepsis	15 (63)	35 (54)	0.631	9 (64)	11 (69)	1	6 (60)	24 (49)	0.731
Heart failure	4 (17)	14 (22)	0.769	3 (21)	0	0.090	1 (10)	14 (29)	0.427
COPD exacerbation	6 (25)	7 (11)	0.104	3 (21)	2 (13)	0.642	3 (30)	5 (10)	0.126
Postoperative respiratory insufficiency	2 (8)	3 (5)	0.609	1 (7)	0	0.467	1 (10)	3 (6)	0.535
Intensive care unit stay and length of hospitalization									
Surgical ICU	4 (17)	7 (11)	0.475	1 (7)	1 (6)	1	3 (30)	6 (12)	0.17
Medical ICU	20 (83)	58 (89)	0.475	13 (93)	15 (94)	1	7 (70)	43 (88)	0.17
Total days ICU	16 [10–28]	26 [17–32]	*0.009*	17 [6–31]	25 [16–32]	0.328	15 [12–17]	26 [17–32]	*0.006*
Death on ICU	12 (50)	25 (38)	0.344	6 (43)	6 (38)	1	6 (60)	20 (34)	0.311
Total days hospital	26 [17–38]	41 [26–56]	*0.004*	31 [19–43]	38 [26–51]	0.176	24 [16–34]	42 [26–56]	*0.008*
Death in hospital	13 (54)	30 (46)	0.634	7 (50)	7 (44)	1	6 (60)	23 (47)	0.506

The baseline data of the entire cohort and subgroups is listed in the corresponding column as median [interquartile range] or as absolute number (%). *p* values were calculated using the Mann-Whitney U test for medians or Fisher’s exact test for categorical data comparing untreated to antivirally treated patients (significant values are indicated in italics, < 0.05). *Abbreviations: HSV* herpes simplex virus, *COPD* chronic obstructive pulmonary disease, *ICU* intensive care unit, *BAL* bronchoalveolar lavage fluid, *TBS* tracheobronchial secretions

Norepinephrine use was recorded as micrograms/kilogram body weight/minute. The following parameters were calculated: partial pressure arterial oxygen and fraction of inspired oxygen (PaO_2_/FiO_2_) ratio, alveolar-arterial O_2_ gradient ([677 mmHg × FiO_2_ − (PaCO_2_/0.8)] − PaO_2_), and lung compliance (tidal volume/[plateau pressure − positive endexpiratory pressure]) [23, 24]. In cases with more than one recorded value per day in the chart, we used the 24-h arithmetic mean for analysis.

### Statistical analysis

Statistical calculations were performed in R (version 3.5.0) [25] with the following external packages: exactRankTests, survival, survminer, ggplot2, and PMCMRplus [26–30]. The nonparametric Mann-Whitney *U* test and Fisher’s exact test were used because data was not normally distributed. The Kaplan-Meier curves and the log-rank test were used to analyze ICU survival. We then applied a multivariable Cox regression model for the effect of acyclovir on ICU survival time, adjusted for age, sex, and SOFA score at d0. The Cox regression model was tested for its proportional hazard assumption via the Schoenfeld residuals tests. Additional models and a propensity score analysis were calculated as shown in Additional file 1: Table S2. The propensity score was determined in a logistic regression model with acyclovir treatment as response variable and age, sex, and SOFA score as predictor covariates.

Daily medians of norepinephrine doses and PaO_2_/Fio_2_ ratios were analyzed for all patients without missing values within 7 days from d0 using the nonparametric Friedman test. This was followed by post hoc analysis by the Friedman-Conover test with *p* value adjustment by false discovery rate (fdr).

The radiographic score and the descriptive part of the LIS score were analyzed by the Wilcoxon signed-rank test comparing the last available X-ray or CT before d0 and the maximum change within a time span of 3 to 15 days.

## Results

### Patient selection and baseline parameters

We tested respiratory secretions of 425 ICU patients for HSV-1/2 replication by PCR. Of these, 126 (29.6%) tested positive for at least 10^3^ HSV-1 copies/mL. Only one patient had additional HSV-2 replication (Fig. 1). All but two patients had clinical signs of pneumonia (elevated C-reactive protein, leukocytosis, purulent tracheal secretions, pulmonary infiltrates, or typical signs in bronchoscopy). Strikingly, 20/50 (40%) low viral load patients responded to culture-guided antibiotic treatment, in contrast to 8/67 (12%) of high viral load patients (*p* < 0.001). These antibiotic responders as well as the two patients without clinical signs of pneumonia were excluded from analysis, so that 89 patients with antibiotic refractory VAP remained: 30 with low viral load (10^3^–10^5^ copies/mL) and 59 with high viral load (> 10^5^ copies/mL). Additional file 1: Table S1 shows microbiology culture results and antibiotics received by these patients; all were treated with at least one antibiotic active against the bacteria identified. A significantly higher proportion of high load patients received antiviral treatment (83% vs 57%, *p* = 0.005, Fig. 1).

The time from VAP diagnosis (detection of infiltrates) to HSV detection was not significantly different between untreated and acyclovir treated patients (Table 1), nor did it correlate with viral load (Additional file 1: Figure S1).

In general, tracheobronchial secretions tended to have higher HSV viral loads than bronchoalveolar lavages, although this difference reached statistical significance only in the entire cohort (*p* = 0.026, Additional file 1: Figure S2a); it was not significant in the subgroups of high and low load patients, respectively (Additional file 1: Figure S2b, c).

As shown in Table 1, antiviral-treated patients had higher HSV loads (median 1.1 × 10^6^ vs 1.3 × 10^4^, *p* < 0.001) especially in bronchoalveolar lavage fluid (median 1.1 × 10^6^ vs 0.9 × 10^4^, *p* < 0.001) and longer total ICU and hospital stays (mean 26 vs 16 days, *p* = 0.009, and 41 vs 26 days, *p* = 0.004, respectively) than untreated patients. During the entire ICU stay, they had longer antibiotic treatment (18 vs 14.5 days, *p* = 0.046); had a greater number of different antibiotic classes (5.0 vs 3.5, *p* = 0.025), particularly ceftazidime (18% vs 0%, *p* = 0.031); and were intubated longer (13 vs 9 days, *p* = 0.017). On the other hand, treated patients had less chronic obstructive pulmonary disease (20% vs 50%, *p* = 0.008) and required lower norepinephrine doses on d0 than untreated patients (median 0.001 vs 0.101 μg/kg body weight/min, *p* = 0.009).

Among the subgroup with high viral load, those receiving antiviral treatment had higher HSV loads (median 3.1 × 10^6^ vs 2.8 × 10^5^, *p* < 0.001) and longer total ICU and hospital stays (26 vs 15 days, *p* = 0.006, and 42 vs 24 days, *p* = 0.008, respectively) than untreated patients. They also received longer antibiotic courses (median 17 vs 12 days, *p* = 0.045) than untreated patients. None of these differences were significant in the subgroup with low viral load.

### Survival time and Cox regression analysis

In the entire cohort of 89 patients with detectable respiratory HSV replication, acyclovir had no significant impact on ICU survival (Fig. 2a). Upon subgroup analysis, however, patients with high viral load who received acyclovir had significantly longer median ICU survival compared to patients without antiviral treatment (22 vs 8 days, *p* = 0.014; Fig. 2c), while no significant difference was demonstrated in low viral load patients (Fig. 2b). To correct for possible confounding, a multiple Cox regression model was adjusted for age, sex, and SOFA score (Fig. 3). The Cox model key assumption of proportional hazards was not violated as tested with the Schoenfeld residuals tests. In all patient groups, higher SOFA scores were consistently associated with increased hazard ratios for ICU death (Fig. 3a–c). The impact of acyclovir on survival remained significant only in high viral load patients (hazard ratio for ICU death 0.31, 95% CI 0.11–0.92, *p* = 0.035; Fig. 3c). Additional Cox models adjusted for different covariates (SOFA, APACHE II, COPD, HSV viral load as continuous variable) and a propensity score analysis gave essentially identical results (see Additional file 1: Table S2). Absolute ICU mortality, however, did not differ between untreated and acyclovir treated patients in the entire cohort (12/24 vs 26/65, *p* = 0.344), the low viral load subgroup (6/14 vs 6/16, *p* = 1), or the high viral subgroup (6/10 vs 20/49, *p* = 0.311).

### Impact of concomitant cytomegalovirus reactivation on ICU survival

Overall, 66 of 89 patients were tested for CMV replication together with HSV. CMV replication could be detected in 14/66 (21%) patients but had no effect on median ICU survival (Additional file 1: Figure S4).

### Changes in clinical and laboratory parameters over the course of antiviral treatment

Upon antiviral treatment (Fig. 4), high viral load patients improved in terms of circulatory support (from day 0 to 6 decrease in mean norepinephrine doses from 0.05 to 0.02 μg/kg body weight/min, fdr-adjusted *p* = 0.049; Fig. 4a) and pulmonary oxygenation (median PaO_2_/FiO_2_ ratio increased during treatment from 187 on day 3 to 241 on day 7, fdr-adjusted *p* = 0.02; Fig. 4b). Pulmonary and circulatory function of untreated patients remained unchanged. Finally, we evaluated all chest X-rays and CTs from high viral load patients. Scores of the first radiograph were slightly worse in patients who subsequently received antiviral treatment than in patients who did not (median 11 vs 8 points, *p* = 0.10; Fig. 4c), but they improved significantly during the course of treatment (11 vs 10 points, *p* < 0.001). The radiographic score did not change in untreated patients for whom at least two sequential X-rays were available for analysis.

## Discussion

The role of HSV as a causative agent of VAP is subject of active debate, since HSV replication is not infrequent in ICU patients (29.6% in our cohort). Some studies have shown that HSV replication is of clinical importance: Ong et al. reported a prevalence of HSV viremia in 26% of septic shock patients admitted to the ICU [31] and Luyt et al. published that 21% of prolonged ventilated patients with clinical deterioration had histologically proven HSV bronchopneumonitis [14]. Identification of these patients under conditions of routine clinical care, however, is challenging, and a clinical algorithm would be helpful.

We suggest testing all patients with a diagnosis of VAP who do not respond to antibiotics for HSV replication in respiratory secretions. We adopted the suggested cutoff of 10^5^ copies/mL [7], and several lines of evidence support this: First, more patients with lower viral loads responded to antibiotics than patients with higher viral loads, suggesting the former may have mostly bacterial causes of pneumonia. Second, the cutoff clearly separated patients responding from those not responding to acyclovir, in both uni- and multivariable analyses. This suggests that high-grade HSV replication may be causative for VAP in patients without other identifiable causes.

Our study is one of the few in which patients with a high likelihood of viral VAP have been identified before testing for HSV replication. Luyt et al. prospectively analyzed 201 ICU patients who were ventilated for more than 5 days, deteriorated clinically, and in whom active lung disease was suspected [14]. These authors found that the presence of histologic or cytologic signs of HSV bronchopneumonitis was associated with higher HSV viral loads. They could, however, not demonstrate any effect of acyclovir treatment on clinical courses or outcomes.

Several previous studies which examined the impact of acyclovir treatment on survival analyzed ICU mortality at one particular time point [14–16]. Since the risk of non-HSV-associated, ICU-acquired complications increases with the length of ICU stay, a survival benefit caused by acyclovir may be underestimated by analyzing mortality after a longer period of time. We found that patients receiving acyclovir had significantly longer ICU stays and a longer duration of mechanical ventilation than untreated patients. We think that this is a consequence of shorter ICU survival of untreated patients, naturally resulting in a numerically reduced duration of ICU stay or mechanical ventilation.

To date, only one study has demonstrated a similar survival benefit of acyclovir, in 29 ICU patients with positive HSV-1 by culture (PCR was not performed) compared to 21 untreated patients (ICU mortality 21% vs 48%, hospital mortality 28% vs 62%) [17]. Although the patients receiving acyclovir treatment, like in our study, had longer ICU stays (55 vs 31 days), and, therefore, had longer risk exposure to other ICU-related complications, acyclovir treatment still was associated with longer survival. This suggests that the impact of acyclovir treatment on survival may be substantial if proper patient selection was performed.

Since acyclovir was associated with a significant longer time to ICU death in high load HSV patients, we expected that recovery of organ dysfunction should also occur during the course of treatment. Indeed, circulatory support, pulmonary oxygenation function, and radiologic signs of pneumonia improved during treatment, but not in untreated patients. Since patients who quickly improved upon antiviral treatment often did not have had a second chest radiograph taken, we likely underestimated radiographic improvement.

However, the following limitations should be considered: First, our study was retrospective and as such cannot prove that acyclovir was causative for our patients’ improvement. However, we think that the effect of acyclovir treatment on survival was surprisingly clear, and the improvement in circulatory and pulmonary oxygenation function was quite impressive.

Second, the group of untreated high load patients is quite small. This poses the question why these patients have not been treated. It should be kept in mind that this is a retrospective study of data acquired during routine clinical practice, so the decision to provide or withhold acyclovir treatment was left entirely to the responsible attending physician. In these patients, given the lack of evidence at that time, detection of HSV replication in the respiratory tract was not considered clinically significant by our clinical staff. To minimize selection bias, we determined a propensity score and added it to a multivariable Cox regression model (as shown in Additional file 1: Table S2), but the results remained essentially the same.

Third, we cannot prove that HSV was the causative pathogen leading to VAP in our patients since histopathologic evaluation was not performed. However, we consider it highly likely, because all patients in the final analysis had signs of pneumonia, all other pulmonary pathogens detected were adequately treated by antibiotics, and no patient responded to antibiotic treatment. Additionally, the therapeutic effect of acyclovir suggests a causative role of HSV in high load patients. Unfortunately, there are no randomized trials examining acyclovir as a treatment for VAP. Only one randomized trial exists regarding prophylactic treatment in patients with acute respiratory distress syndrome. Acyclovir reduced HSV detection in viral culture, but did not reduce mortality [32].

Fourth, the day of material sampling was not standardized, but based on clinical symptoms as judged by clinicians and microbiologists. Timing may impact HSV detection, since HSV load may increase with the length of mechanical ventilation [33]. Thus, some of our low viral load patients may have been classified as high load patients if the PCR had been performed later. However, they all had clinical signs of pneumonia at the time of sampling. Moreover, we found no correlation between time from VAP diagnosis to HSV detection and viral load (Additional file 1: Figure S1). This suggests that viral loads may not rapidly increase during several days. Therefore, we consider this time point as clinically adequate and suggest that a few days of variation in PCR diagnosis does not significantly alter the results.

## Conclusions

Taken together, we showed that acyclovir treatment was associated with a significantly longer time to death in the ICU, reduced hazard ratio for ICU death, and improved circulatory and pulmonary oxygenation function in patients with VAP not responding to antibiotic treatment and with high HSV load. We suggest testing all patients with a diagnosis of antibiotic refractory VAP for HSV replication in respiratory secretions and considering acyclovir treatment if more than 10^5^ copies/mL were detected.

## Supplementary information

**Additional file 1: Figure S1.** Time from imaging to PCR vs HSV viral load in all patients with infiltrates (*n* = 78). **Figure S2.** Type of obtaining respiratory secretions and viral load. **Figure S3.** Re-analysis of radiographic findings using the descriptive part of the LIS Score. **Figure S4.** Impact of concomitant cytomegalovirus (CMV) reactivation on survival in HSV patients. **Table S1. a** Microbiology culture results of the respiratory secretions. **b** Summary of antibiotic treatments during ICU stay. **Table S2.** Summary of harzard ratios for ICU death from different calculated COX regression models.

## Data Availability

The datasets used and analyzed during the current study are available from the corresponding author on reasonable request.

## Abbreviations

95% CI: 95% confidence interval
APACHE II: Acute Physiology, Age, Chronic Health Evaluation II Score
CPIS: Clinical Pulmonary Infection Score
CT: Computed tomography
d0: Day 0
fdr: False discovery rate adjustment
FiO_2_: Fraction of inspired oxygen
HSV: Herpes simplex virus
HSV-1: Herpes simplex virus type 1
HSV-2: Herpes simples virus type 2
ICU: Intensive care unit
LIS: Lung Injury Score
PaCO_2_: Arterial partial pressure of carbon dioxide
PaO_2_: Arterial partial pressure of oxygen
PCR: Polymerase chain reaction
SOFA: Sequential Organ Failure Assessment Score
VAP: Ventilator-associated pneumonia
