# Supplementary material for: Impact of acyclovir use on survival of patients with ventilator-associated pneumonia and high load herpes simplex virus replication
Source: Crit Care. 2020 Jan 10;24:12. doi: 10.1186/s13054-019-2701-5 (PMC6954562; doi:10.1186/s13054-019-2701-5)
Supplement: Supplementary file 1 — Additional file 1: Figure S1. Time from imaging to PCR vs HSV viral load in all patients with infiltrates (n = 78). Figure S2. Type of obtaining respiratory secretions and viral load. Figure S3. Re-analysis of radiographic findings using the descriptive part of the LIS Score. Figure S4. Impact of concomitant cytomegalovirus (CMV) reactivation on survival in HSV patients. Table S1. a Microbiology culture results of the respiratory secretions. b Summary of antibiotic treatments during ICU stay. Table S2. Summary of harzard ratios for ICU death from different calculated COX regression models. [file 13054_2019_2701_MOESM1_ESM.doc]

**Supplementary Data for Schuierer et al. , “Impact of acyclovir use on survival of patients with ventilator-associated pneumonia and high load herpes simplex virus replication: a retrospective observational cohort study”**

**
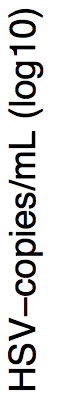
**

**Figure S1 Time from imaging to PCR vs HSV viral load in all patients with infiltrates (n=78)**

One patient with 52 days from the first infiltrates to high viral load PCR was excluded from analysis, because a very low HSV viral load was detected 7 days after the diagnosis of atypical pneumonia. The linear regression model showed no significant correlation between time to PCR and viral load (p=0.130, R^2^ =0.174).

**Figure S2 Type of obtaining respiratory secretions and viral load**

Viral load was compared between bronchoalveolar lavage fluids (BAL) and tracheobronchial secretions (TBS). Tracheobronchial secretions tended to have higher viral loads than bronchoalveolar lavages; although this difference reached statistical significance only in the entire cohort, it was not significantly different in the subgroups of high and low viral load. a) Entire cohort. b) Subgroup with low viral load (10^3^-10^5^ HSV copies/mL) c) Subgroup with high viral load (>10^5^ HSV copies/mL).

**a)**

**b)**

**c)**

**Figure S4 Impact of concomitant cytomegalovirus (CMV) reactivation on survival in HSV patients**

Day 0 (d0) was defined as the date of first detection of significant HSV-1/2 replication in untreated patients or as the date of acyclovir treatment start for patients receiving acyclovir. + = censored. The p-value was calculated using a log-rank test.

**Figure S3 Re-analysis of radiographic findings using the descriptive part of the LIS Score**

The same radiographic imaging was re-evaluated as already shown for radiographic score in Fig. 4b. Results are essentially in line to our score that seems to underestimate worsening of radiographic findings in untreated patients, while improvement in treated patients is detected to the same extent. Day 0 (d0) was defined as the date of first detection of significant HSV-1/2 replication in untreated patients or as the date of acyclovir treatment start for patients receiving acyclovir.

| **Table S1a** Microbiology culture results of the respiratory secretions | | |
| --- | --- | --- |
| **pathogen** | **# of patients** |  |
| Staphylococcus aureus | 8 |  |
| Escherichia coli | 4 |  |
| Proteus mirabilis | 3 |  |
| Klebsiella oxytoca | 2 |  |
| Klebsiella pneumoniae | 1 |  |
| Klebsiella aerogenes | 1 |  |
| Haemophilus influenzae | 1 |  |
| Pseudomonas aeruginosa | 1 |  |
| Citrobacter freundii | 1 |  |
| Enterobacter cloacae | 1 |  |
| Streptococcus agalactiae | 1 |  |
| Morganella morganii | 1 |  |
| Serratia marcescens | 1 |  |
| *Mycobacterium tuberculosis | 1 |  |
| In parallel to HSV testing 82/89 patients received microbiologic testing of their respiratory secretions with 22 positive cultures with the shown pathogens (5 patients had two pathogens).  *Accidental finding four weeks post mortem. Initial workup ad admission showed no findings suggestive for tuberculosis. | |  |

| **Table S1b** Summary of antibiotic treatments during ICU stay | | | | |
| --- | --- | --- | --- | --- |
| **Antbiotic classes** | **# of patients** | | **included substances** |  |
| penicillins | 6 | Penicillin, Flucloxacillin | |  |
| penicillins + beta-lactamase inhibitors | 92 | Piperacillin/tazobactam,Ampicillin/sulbactam, Amoxicillin/clavulanate | |  |
| cephalosporins | 47 | Cefazolin, Cefuroxime, Ceftazidime, Ceftriaxone, Cefepime | |  |
| carbapenems | 83 | Meropenem, Imipenem | |  |
| macrolides | 40 | Azithromycin, Clarithromycin, Erythromycin, Roxithromicin | |  |
| aminoglycosides | 5 | Gentamicin, Amikacin | |  |
| quinolones | 42 | Ciprofloxacin, Moxifloxacin, Levofloxacin | |  |
| sulfonamides | 15 | Trimethoprim-Sulfamethoxazole | |  |
| lincosamides | 6 | Clindamycin | |  |
| against multidrug resistant gram-positives | 50 | Vancomycin, Linezolid, Daptomycin, Rifampicin | |  |
| others | 8 | Metronidazole, Doxycycline, Colistin | |  |
| Antibiotic therapy met current society recommendations for treatment of VAP and at least one effective substance was given. Patients often received >1 antibiotic. | | | |  |

| **Table S2** Summary of harzard ratios for ICU death from different calculated COX regression models | | | | |
| --- | --- | --- | --- | --- |
|  | | Acyclovir + |  |  |
|  | | HR | 95% CI | p |
| **All (n=89; 38 events)** | |  |  |  |
|  | Age, Sex, SOFA adjusted | 0.74 | 0.37-1.47 | 0.382 |
|  | Age, Sex, APACHE II adjusted | 0.69 | 0.35-1.38 | 0.300 |
|  | Age, Sex, SOFA adjusted + inverse probability of treatment (propensity score) weighted | 0.69 | 0.39-1.20 | 0.185 |
|  | Age, Sex, SOFA, COPD adjusted | 0.57 | 0.27-1.22 | 0.149 |
|  | Age, Sex, SOFA, HSV viral load adjusted | 0.86 | 0.43-1.73 | 0.666 |
| **Low viral load (n=30, 12 events)** | |  |  |  |
|  | Age, Sex, SOFA adjusted | 0.93 | 0.28-3.13 | 0.910 |
|  | Age, Sex, APACHE II adjusted | 0.93 | 0.29-2.95 | 0.896 |
|  | Age, Sex, SOFA adjusted + inverse probability of treatment (propensity score) weighted | 0.89 | 0.34-2.31 | 0.815 |
|  | Age, Sex, SOFA, COPD adjusted | 0.87 | 0.26-2.89 | 0.816 |
|  | Age, Sex, SOFA, HSV viral load adjusted | 0.51 | 0.11-2.33 | 0.382 |
| **High viral load (n=59; 26 events)** | |  |  |  |
|  | Age, Sex, SOFA adjusted | 0.31 | 0.11-0.92 | 0.035 |
|  | Age, Sex, APACHE II adjusted | 0.28 | 0.10-0.80 | 0.017 |
|  | Age, Sex, SOFA adjusted + inverse probability of treatment (propensity score) weighted | 0.29 | 0.12-0.70 | 0.006 |
|  | Age, Sex, SOFA, COPD adjusted | 0.17 | 0.05-0.61 | 0.007 |
|  | Age, Sex, SOFA, HSV viral load adjusted | 0.33 | 0.11-0.97 | 0.044 |
| The hazard ratios (HR) for ICU death derive from the listed Cox regression models analysing the entire cohort and the two subgroups: In addition to the SOFA score adjusted Cox regression model (as shown in Fig. 3) a separate model with the APACHE score was calculated due to high correlation of both established scores. To minimize the confounding by indication a propensity score was included in a further multiple Cox model using inverse probability of treatment weighting. The last two models were created to adress the unbalanced baseline characteristics “COPD” and “quantitative viral load”, both covariates with an potential impact on survival. | | | | |
